# Ex vivo, microelectrode analysis of conduction through the AV node of wild‐type and *Nkx2‐5* mutant mouse hearts as guided by a Cx40‐eGFP transgenic reporter

**DOI:** 10.14814/phy2.285

**Published:** 2014-04-23

**Authors:** Avihu Z. Gazit, Alex Li, Jacob S. Choi, Lucile Miquerol, Patrick Y. Jay

**Affiliations:** 1 Department of Pediatrics, Washington University School of Medicine, St. Louis, Missouri; 2 Developmental Biology Institute of Marseille, CNRS UMR 7288, Aix Marseille University, Marseille, France; 3 Department of Genetics, Washington University School of Medicine, St. Louis, Missouri

**Keywords:** Atrioventricular node, central conduction system, mouse, Nkx2‐5

## Abstract

Mutations of the cardiac transcription factor *NKX2‐5* cause hypoplastic development of the AV node and conduction block. How the anatomy of the mutant AV node relates to its function is unknown. We thus studied conduction through the AV nodal region in ex vivo preparations of wild‐type and *Nkx2‐5*^+/−^ mouse hearts in which the central conduction system was highlighted by a transgenic Cx40‐eGFP reporter. Fluorescence imaging guided electrode placement and pacing of the inferior and superior approaches to the AV node. *Nkx2‐5*^+/−^ hearts had a prolonged atrio‐His interval compared to the wild type, consistent with previous in vivo observations. The conduction time to the His bundle from the Cx40^−^ AV nodal region that is superior to and immediately adjacent to the Cx40^+^ lower node is slightly, but not significantly greater in *Nkx2‐5*^+/−^ than wild‐type hearts. A novel phenotype was also observed. Pacing the Cx40^−^ inferior approach to the AV node with increasing stimulus strength led to progressive shortening of the stimulus‐to‐His conduction interval in wild‐type but not *Nkx2‐5*^+/−^ hearts. The strength of pacing at the Cx40^−^ superior approach had no effect on the conduction interval in either group. The prolonged AV delay in the *Nkx2‐5*^+/−^ heart appears to arise before the Cx40^+^ lower node. Whether the pacing phenotype explains the mutant's conduction defect is uncertain, but the observation adds to a number of unique properties of the inferior approach to the AV node.

## Abstract (precis)

Mutations of the cardiac transcription factor *NKX2‐5* cause hypoplastic development of the AV node and associated conduction defects. Here, we use microelectrodes to measure conduction intervals between locations in the atrial myocardium and subdomains of the AV node and the His bundle, as guided by Cx40‐eGFP fluorescence imaging. We also report a novel property of pacing the inferior approach to the AV node and its related mutant phenotype.

## Introduction

Mutations of the transcription factor *NKX2‐5* cause cardiac conduction defects in humans and mice (Schott et al. 1998; Benson et al. 1999; Jay et al. 2004). Heterozygous *Nkx2‐5* knockout mice have hypoplastic development and electrophysiologic defects of the AV node, His bundle, and Purkinje system (Jay et al. 2004; Meysen et al. 2007). A thin, hypocellular His bundle plausibly explains a low amplitude His signal, as detected by intracardiac electrogram recordings. Cellular hypoplasia of the Purkinje system explains an intraventricular conduction defect, as demonstrated by the wider QRS interval of the mutant on surface electrocardiograms (Jay et al. 2004). Conditional deletion of *Nkx2‐5* in the ventricular myocyte lineage reveals a similar relationship between conduction system hypoplasia and even higher grade AV block (Pashmforoush et al. 2004).

How hypoplasia of the *Nkx2‐5*^+/−^ AV node relates to its diminished function is less clear. *Nkx2‐5*^+/−^ mice develops an abnormally prolonged AV delay between 4 and 7 weeks of age. Surface electrocardiograms and intracardiac electrophysiologic studies indicate that the abnormal prolongation occurs at or after the border of the atrial myocardium and AV node but before the His bundle (Jay et al. 2004). The murine central conduction system contains multiple anatomic domains that express a combination of markers, including Cx40, Cx45, and Hcn4 (Aanhaanen et al. 2010). The compact AV node and inferior nodal extension, which are Cx40^−^, Cx45^+^, and Hcn4^+^, is much smaller or absent in the *Nkx2‐5*^+/−^ heart compared to the wild type (Jay et al. 2004; Risebro et al. 2012). The lower AV node and His bundle, which are Cx40^+^, Cx45^+^, and Hcn4^+^, are also smaller in the mutant. To determine where the delay arises relative to these subdomains and the atrial myocardium, we dissected hearts from wild‐type and *Nkx2‐5*^+/−^ mice that expressed enhanced green fluorescence protein (eGFP) under the control of the Cx40 locus. The fluorescent reporter highlights the atrial myocardium and Cx40^+^ conduction cells from the lower AV node to the Purkinje system (Miquerol et al. 2004).

Conduction from the atrium proceeds through the Cx40^−^ superior or inferior approach, to the Cx40^−^ compact AV node, and then to the Cx40^+^ lower node and His bundle (Medkour et al. 1998; Khalife et al. 1999; Zhang et al. 2003; Hucker et al. 2007). In the present experiments, the central conduction system was exposed in a superperfused, ex vivo preparation. Two electrodes were placed to measure conduction intervals from a roaming electrode to the His bundle. Guided by Cx40‐eGFP fluorescent imaging, the roaming electrode could be placed on the atrial myocardium, at the Cx40^−^ inferior or superior approaches, or on the Cx40^+^ lower node. As the compact AV node is also Cx40^−^, it was not possible to determine exactly the boundary between the inferior or superior approach and the node itself, thus limiting the resolution of mapping within a small region. The results indicate that the prolonged AV delay in *Nkx2‐5*^+/−^ mice arises before the Cx40^+^ lower node and reveal a novel property of pacing at the inferior nodal approach. To our knowledge, this is the first example in which conduction system‐specific GFP reporter has been used to guide electrophysiologic mapping studies.

## Methods

### Mouse strains

Connexin40 (Cx40^eGFP^) knockout mice, in which eGFP replaces the Cx40 coding sequence, were crossed to *Nkx2‐5*^+/−^ mice. Wild‐type and *Nkx2‐5*^+/−^ offspring that were heterozygous for the Cx40^eGFP^ allele were studied. The strains have been previously described (Jay et al. 2004; Miquerol et al. 2004). Animals were housed under standard conditions. The experiments were approved by the animal studies committee at Washington University School of Medicine.

### Preparation of hearts for study ex vivo

Electrophysiologic studies were performed on superperfused ex vivo preparations. The mice, ages 4 or 7 weeks, were anesthetized with 100 mg/kg pentobarbital sodium and anticoagulated with 100 IU intraperitoneal heparin. The heart was excised, and the aorta was cannulated for perfusion at a pressure of 30 mmHg for 3 min with ice‐cold, oxygenated (95%O_2_–5%CO_2_) Tyrode solution (mmol/L: 128.2 NaCl, 4.7 KCl, 2.0 CaCl_2_, 1.0 MgCl_2_, 20 NHCO_3_, 0.7 NaH_2_PO_4_, 11.1 dextrose, pH 7.4). CaCl_2_ and dextrose were added the day of the experiment. The heart was then decannulated and immersed in a dissection chamber filled with ice‐cold, oxygenated Tyrode solution. The apex was removed, and the right ventricular free wall, tricuspid valve annulus, and right atrial free wall were cut to expose the central conduction system. The preparation was then superperfused with oxygenated Tyrode solution at 37°C.

### Electrophysiologic study of hearts ex vivo

**Figure 1.**
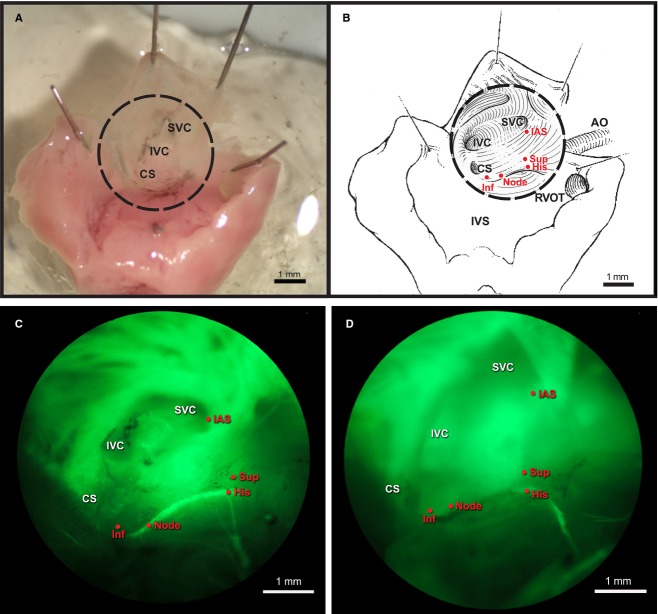
(A) The central conduction system is exposed in a superperfused, ex vivo preparation. (B) Schematic diagram of the anatomical landmarks and electrode recording and pacing sites. Electrode recording and pacing sites are shown at the interatrial septum (IAS), superior (Sup) and inferior (Inf) approaches, Cx40^+^ node and His bundle. (C) Imaging of a Cx40^eGFP^ reporter reveals the fluorescent atrial myocardium, Cx40^+^ portion of the AV node and the His bundle as well as the Cx40^−^ inferior and superior approaches to the AV node. The Cx40^+^ portion of the central conduction system is larger in (C) wild‐type than (D) *Nkx2‐5*^+/−^ hearts. Ao, aorta; CS, coronary sinus; IVC, inferior vena cava; IVS, interventricular septum; RVOT, right ventricular outflow tract; SVC, superior vena cava.

Two platinum/iridium concentric bipolar electrodes were employed for pacing and recording (FHC Inc., Bowdoin, ME). The electrode's outer and inner pole diameters were 125 and 25 *μ*m, respectively. The recording electrode was placed on the His bundle and remained in place for the duration of an experiment. A roaming electrode was used to detect the intrinsic rhythm and to pace at the interatrial septum, the inferior and superior approaches to the AV node and the node itself, as defined by Cx40‐eGFP fluorescence (Fig. 1). The pacing threshold for capture was determined at each site. The time interval for conduction from the stimulated site to the His bundle was determined at twice the threshold while pacing at a cycle length of 128 msec. Signals were recorded on a custom‐built multichannel data acquisition system. The signals were recorded at a gain of 1000 with a frequency response of 50–1000 Hz and digitized (12 bit A/D) at 2000 Hz.

For pacing at the inferior and superior approaches, the relationship between stimulus strength and the conduction interval was assessed by increasing the output in stepwise increments. The dependence of conduction interval on stimulus strength was determined by linear regression and a *t*‐test on the slope.

### Isoproterenol challenge

Limb lead ECGs were obtained in 7‐week‐old *Nkx2‐5*^+/−^ and WT mice. The mice were anesthetized with intraperitoneal administration of ketamine (0.033 mg/gm) and pentobarbital (0.033 mg/gm). Blow‐by oxygen was administered. The level of anesthesia was deemed adequate by the absence of withdrawal to pinching of the hindlimb. Normal saline was administered by intraperitoneal injection first, followed by isoproterenol (0.5 mcg/gm).

ECGs were recorded at a frequency response of 0.5–125 Hz on a Gould ECG 5900 signal conditioner system. Data were digitized (12 bit A/D, 1000 Hz) and stored on a custom‐built data acquisition and analysis system.

### Statistical analysis

Numerical data are presented as mean ± SEM. Statistical significance was determined by a *P*‐value < 0.05 in two‐sided *t*‐tests.

## Results

### Visualization of AV conduction system in ex vivo preparations

The Cx40^eGFP^ allele permits visualization of the central conduction system in live preparations. The atrial myocardium also expresses Cx40, but a region between it and the Cx40^+^ central conduction system does not (Fig. 1). As previously reported (Jay et al. 2004), the AV node and His bundle are hypoplastic in *Nkx2‐5*^+/−^ hearts compared to the wild type. With GFP imaging and anatomic landmarks to guide us, we could pace the Cx40^−^ superior (Sup) or inferior (Inf) approach to the AV node or the lower Cx40^+^ AV node/His bundle (Node) directly (Fig. 1).

### Atrio‐His conduction intervals are abnormally prolonged in the *Nkx2‐5*^*+/−*^ heart ex vivo

**Figure 2.**
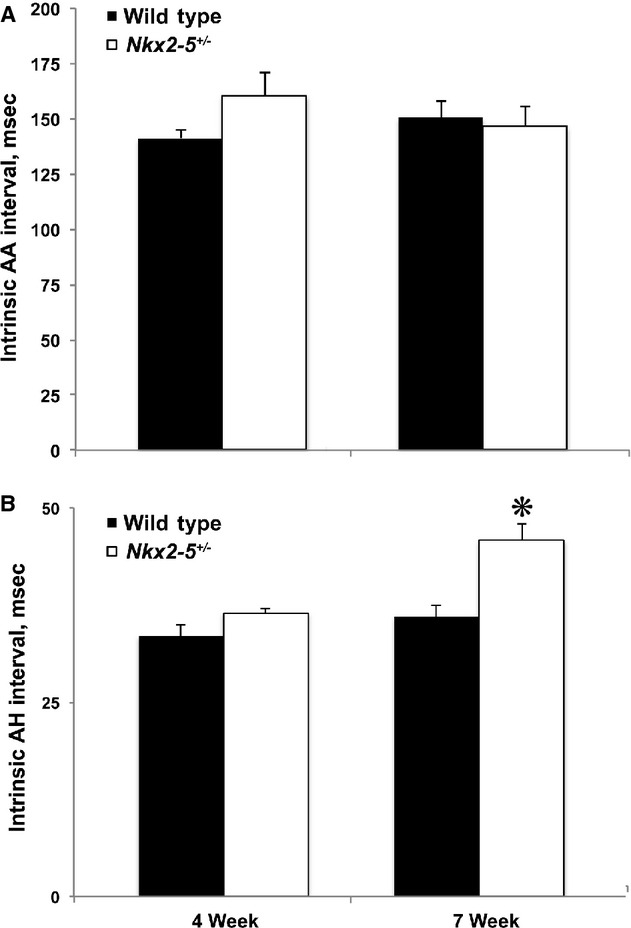
The atrial (AA) and atrial‐His (AH) intervals during spontaneous rhythm were recorded prior to pacing experiments. (A) *Nkx2‐5*^*+/*−^ and wild‐type preparations revealed similar AA intervals regardless of the age of the mouse. (B) AH intervals were similar in 4‐week‐old *Nkx2‐5*^+/−^ and wild‐type mice but longer in 7‐week‐old *Nkx2‐5*^*+/*−^ mice compared to wild type, consistent with previously reported in vivo results. *N* = 3 *Nkx2‐5*^+/−^ and 12 wild type at 4 weeks. *N* = 9 *Nkx2‐5*^+/−^ and 18 wild type at 7 weeks. *P = 0.0025.

*Nkx2‐5*^+/−^ mice develop abnormal prolongation of the PR and atrio‐His (AH) intervals between 4 and 7 weeks of age, as determined by surface ECGs and in vivo intracardiac electrophysiologic recordings (Jay et al. 2004). Similar observations were obtained from ex vivo preparations of 4‐ or 7‐week‐old mouse hearts. Microelectrodes were placed on the interatrial septum and His bundle to measure the heart rate, that is, AA, and AH intervals. During spontaneous rhythm, the AA intervals of *Nkx2‐5*^+/−^ and wild‐type hearts in both age groups were equivalent (Fig. 2A). At 4 weeks of age, the AH intervals of the mutant and wild type were the same. At 7 weeks, the *Nkx2‐5*^+/−^ AH interval was significantly prolonged compared to the wild type (45.8 ± 2.2 ms vs. 36.0 ± 1.6 ms, *P* = 0.0025; Fig. 2B).

**Figure 3.**
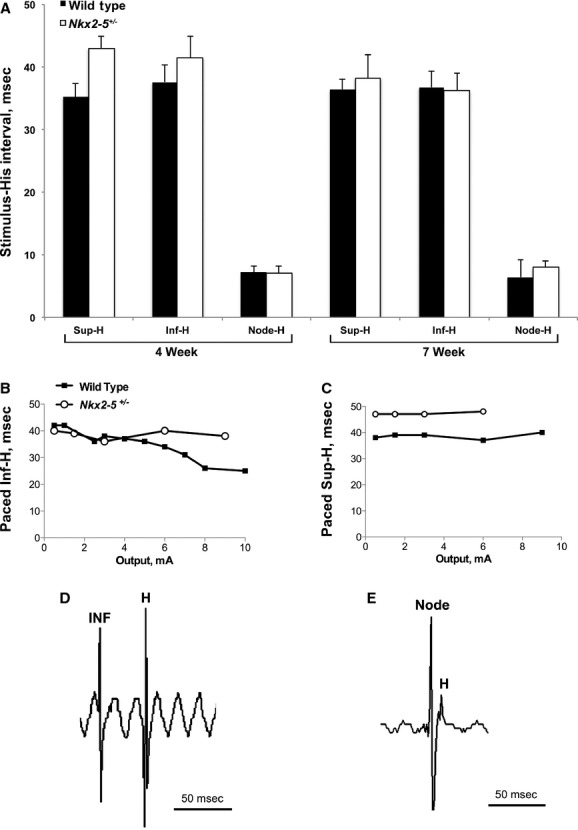
Pacing of the Cx40^−^ superior and inferior approaches to the AV node and the proximal Cx40^+^ portion of the node was performed to measure the Sup‐H, Inf‐H, and Node‐H conduction intervals. The recording electrode remained fixed on the His bundle. (A) The intervals were measured in 4‐ and 7‐week‐old *Nkx2‐5*^*+/*−^ and wild‐type mouse hearts at twice the depolarization threshold for a preparation (1.0 ± 0.1 mAmp at either Cx40^−^ approach and 0.8 ± 0.1 mAmp at the Cx40^+^ node in every experimental group, mean ± SEM). The Sup‐H interval tended to be longer in *Nkx2‐5*^+/−^ hearts compared to wild type. *Nkx2‐5*^+/−^ and wild‐type hearts had similar Inf‐H and Node‐H intervals. *N* = 4 *Nkx2‐5*^+/−^ and 5 wild type at 4 weeks. *N* = 4 *Nkx2‐5*^+/−^ and 7 wild type at 7 weeks. (B) The Inf‐H interval shortens as the strength of the pacing stimulus is increased in all of 10 wild‐type but none of 8 *Nkx2‐5*^+/−^ hearts. Shortening does not reflect capture of lower nodal tissue because Inf‐H is much greater than Node‐H. Representative examples are shown. (C) The Sup‐H interval does not depend upon the strength of the pacing stimulus in either genotypic group. (D, E) Representative traces are shown for pacing at an approach to the AV node, in this example the Cx40^−^ inferior approach, and the Cx40^+^ lower node.

*Nkx2‐5*^+/−^ mice have a significantly prolonged AH interval, but the conduction intervals from a pacing stimulus delivered at the Cx40^−^ superior AV nodal approach to the His bundle, that is, Sup‐H, only tend to be slightly greater in the mutant at either 4 or 7 weeks of age (Fig. 3A). Pacing at the Cx40^−^ inferior approach revealed similar Inf‐H intervals between wild‐type and *Nkx2‐5*^+/−^ mice at either 4 or 7 weeks of age (Fig. 3A). The conduction velocity from the Cx40^+^ lower node to the His bundle, as indicated by the Node‐H interval, was also equivalent. As the Node‐H interval comprises a minor fraction of the AV delay, the prolonged AH interval associated with *Nkx2‐5* haploinsufficiency arises before the Cx40^+^ lower node (Fig. 3A). Unfortunately, the data do not allow us to localize more precisely where the abnormally prolonged AH interval arises.

### A novel property of conduction from the inferior AV nodal approach to the His bundle

In the course of mapping conduction through wild‐type and *Nkx2‐5*^+/−^ hearts, we discovered a novel property and mutant phenotype associated with pacing the inferior approach to the AV node. To determine the depolarization threshold in the Cx40^−^ region immediately adjacent to the Cx40^+^ lower node, the stimulus output was incrementally increased to the threshold and then beyond. The depolarization thresholds were similar at the superior and inferior approaches in *Nkx2‐5*^+/−^ and wild‐type hearts.

In wild‐type hearts, the Inf‐H interval decreased as the strength of the pacing stimulus was increased (Fig. 3B). The dependence of conduction velocity on stimulus strength was observed in 4‐ and 7‐week‐old mice. Shortening of the Inf‐H interval at higher outputs is not due to capture of nodal tissue or His bundle because the Cx40^+^ Node‐H conduction time is much shorter, that is, <10 msec (Fig. 3A). Notably, in *Nkx2‐5*^+/−^ hearts, the Inf‐H interval remained constant as a function of the pacing stimulus strength (Fig. 3B). The patterns were consistent: the dependence of the Inf‐H interval on stimulus output was observed in all 10 wild‐type but none of eight *Nkx2‐5*^+/−^ hearts. The strength of the pacing stimulus had no effect on conduction at the superior approach in either wild‐type or *Nkx2‐5*^+/−^ hearts (Fig. 3C).

The absence of shortening of the Inf‐H interval in *Nkx2‐5*^+/−^ hearts is not related to an absolute inability to shorten the PR interval. For example, adrenergic stimulation accelerates heart rate and atrioventricular conduction. Wild‐type and *Nkx2‐5*^+/−^ mice demonstrated similar shortening of the RR and PR intervals in response to isoproterenol relative to the baseline (data not shown). Normal saline had no effect on the intervals in either group.

## Discussion

Mutations of the cardiac transcription factor NKX2‐5 cause varying degrees of AV block (Schott et al. 1998; Benson et al. 1999; Jay et al. 2004; Pashmforoush et al. 2004). Common variants of *NKX2‐5* influence the PR interval and the risk of atrial fibrillation; the latter association has also been reported in the *Nkx2‐5*^+/−^ mouse (Tanaka et al. 2002; Pfeufer et al. 2010). The mechanistic basis of the AV block is unknown, but the Cx40^−^/Cx45^+^/Hcn4^+^ domain corresponding to the compact AV node and inferior nodal extension is known to be hypocellular in *Nkx2‐5*^+/−^ mice (Jay et al. 2004; Aanhaanen et al. 2010; Risebro et al. 2012). We thus examined conduction through the AV nodal region in ex vivo preparations of wild‐type and *Nkx2‐5*^+/−^ hearts. Electrode mapping suggests that the abnormal prolongation of the AH interval in *Nkx2‐5*^+/−^ hearts arises before the Cx40^+^ lower node. Although *Nkx2‐5* haploinsufficiency causes a paucity of Cx40^+^ cells from the lower AV node to the Purkinje system, the intrinsic function of the cells appears normal by a number of measures, including the Node‐H intervals reported here. In vivo intracardiac electrograms reveal normal conduction velocity from the His bundle to the distal Purkinje system in *Nkx2‐5*^+/−^ mice, and patch clamp experiments reveal normal action potentials in *Nkx2‐5*^+/−^ Purkinje cells (Jay et al. 2004; Meysen et al. 2007).

We could not determine where proximal to the lower Cx40^+^ AV node the abnormal AH delay arises in *Nkx2‐5*^+/−^ mice. The relative imprecision of manual electrode placement makes it difficult to map conduction between the atrial myocardium and Cx40^+^ lower AV node. Higher spatiotemporal resolution, as offered by optical mapping (Dobrzynski et al. 2003; Hucker et al. 2007), seems necessary to determine more precisely where the prolonged delay arises.

We observed a novel property of pacing the inferior approach to the AV node and a new *Nkx2‐5* mutant phenotype. The inferior nodal extension has a couple intriguing characteristics, as revealed by studies of ex vivo rabbit preparations. First, atrioventricular junctional rhythm usually originates from the inferior nodal extension (Dobrzynski et al. 2003). Second, conduction from the inferior nodal extension to the His bundle appears to bypass the compact AV node – the intrinsic AV delay associated with conduction through the superior approach or fast pathway is absent when the inferior approach or slow pathway is directly stimulated (Zhang et al. 2001; Hucker et al. 2007). We show here that higher pacing stimulus outputs at the Cx40^−^ inferior approach to the AV node are associated with shorter stimulus‐to‐His conduction intervals in wild‐type but not *Nkx2‐5*^+/−^ mouse hearts. There is no dependence of conduction interval on pacing stimulus strength at the Cx40^−^ superior approach in either group. Efimov and colleagues have suggested that the inferior nodal extension could serve as an alternative, larger pacing site to achieve His bundle depolarization (Hucker et al. 2007). If our observation in the mouse extends to larger mammals, one can imagine modulating the AV delay by varying the output of pacing at the inferior approach to the AV node.

The *Nkx2‐5*^+/−^ AV conduction defect is probably not explained by the absence of a dependence of the Inf‐H interval on stimulus strength. The absence is observed in 4‐week‐old *Nkx2‐5*^+/−^ hearts, whereas the quantitative, abnormal delay arises between ages 4 and 7 weeks. We cannot rule out a mechanistic relationship, however, because we did not assess the route of conduction or intrinsic stimulus strength at the approaches to the AV node during normal sinus or atrial‐paced rhythm.

One may wonder whether the novel property could be related to slow and fast pathway physiology. Conduction from the atrium to the His bundle proceeds via the fast or slow pathway, which are located at the superior or inferior approach to the AV node, respectively (Medkour et al. 1998; Khalife et al. 1999; Zhang et al. 2003; Hucker et al. 2007). The pathways are typically described by their differential conduction delays and refractory periods, as elicited by premature stimuli delivered to the atrial myocardium. Atrial extrastimulus testing has demonstrated slow and fast pathway physiology in the mouse, but the anatomy of the pathways has not been characterized as has been in larger mammals (Maguire et al. 2000). A large number of genes have been discovered in the mouse to affect the specification and patterning of the conduction system (Munshi 2012). In combination with high‐resolution methods like optical mapping, the mouse might thus be used to dissect the complex molecular and structural basis of AV nodal physiology.

## Acknowledgments

We thank Kathryn Yamada and Richard Schuessler for sharing their equipment and expertise.

## Conflict of Interest

The authors have no financial conflicts of interest.
